# Behaviors and Knowledge Related to Intentional Outdoor Tanning Among Adults in the Western Region of Saudi Arabia

**DOI:** 10.7759/cureus.33140

**Published:** 2022-12-30

**Authors:** Manal O Alsulami, Abdullah F Albadri, Zeyad A Helmy, Sahar S Alsifri, Mohammed F Bondagji, Amjad M Alshehri, Esraa A Alzahrani

**Affiliations:** 1 Faculty of Medicine, King Abdulaziz University, Jeddah, SAU; 2 Dermatology, King Fahad General Hospital, Jeddah, SAU; 3 Faculty of Medicine, Umm Al-Qura University, Makkah, SAU; 4 Faculty of Medicine, King Saud Bin Abdulaziz University for Health Sciences, Jeddah, SAU

**Keywords:** skin cancer, photoaging, uv, outdoor tanning, dermatology

## Abstract

Background: Intentional outdoor tanning is the process through which people expose their skin to ultraviolet (UV) radiation to darken their skin color toward brown or bronze. UV radiation is a well-known modifiable risk factor for photoaging, photoallergic reactions, and phototoxicity.

Objective: The aim of this study was to assess the behaviors and knowledge of intentional outdoor tanning among adults in the western region of Saudi Arabia.

Methods: A cross-sectional study was conducted among 1023 adults from the general population who met the eligibility criteria. Data were collected in October and November 2022 using an electronic questionnaire to assess the sociodemographic data, behaviors, and knowledge of the participants related to intentional outdoor tanning.

Results: Overall, 24.5% of the participants intentionally tanned outdoors, the majority of which (203, 80.9%) were exposed to the sun for more than 20 minutes. The participants who intentionally tan outdoors were significantly younger than those who do not (32 ± 10.7 vs. 38.4 ± 13.9, respectively, P <0.001). Most of the participants had good knowledge of the disadvantages of sun exposure on the skin (61.3%). Additionally, good knowledge was significantly more prevalent in women (70.3%) than men (48.6%) (P <0.001).

Conclusion: Individuals who intentionally tan outdoors engage in other behaviors or beliefs that increase exposure to UV rays. This points to the need for comprehensive interventions such as community-counseling campaigns to address these new trends and their relationship with photoaging and skin cancer.

## Introduction

Intentional outdoor tanning is the process through which people expose their skin to ultraviolet (UV) radiation to darken their skin color toward brown or bronze. UV radiation is a well-known modifiable risk factor for photoaging, photoallergic reactions, phototoxicity, and carcinogenesis, including the development of life-threatening melanomas.

Overexposure to UV radiation is thus a public health concern [1,2]. Approximately three million non-melanoma skin cancers and 1000 melanoma cases are diagnosed globally every year [3]. Skin cancer is more common in the white population due to their high sensitivity to exposure to UV radiation [4]. However, this malignant disease can also have a major health impact on Arab countries, especially in desert areas. Occupational and recreational exposure as well as the duration of exposure to UV both impact the prevalence of skin cancer [5-7]. In accordance with the most recent Saudi cancer registration, skin tumor was 3.2% among all recently discovered tumor cases in 2010 [8].

UV exposure is essential to protect against bone diseases such as rickets, osteomalacia, and osteoporosis, as it plays a role in vitamin D synthesis. It is also used by dermatologists to treat several dermatological conditions such as psoriasis and vitiligo.

However, chronic unnecessary sun exposure can increase the risk of sun-related adverse outcomes for individuals with normal skin [9]. Modifying the tanning perception among the population may play an important role in behavioral changes that could help decrease these adverse outcomes [10]. The aim of this study is to assess the behaviors and knowledge of intentional outdoor tanning among adults in the western region of Saudi Arabia.

## Materials and methods

Study design and data collection

This observational, cross-sectional study was conducted in October and November 2022 in the western region of Saudi Arabia. An online self-administered questionnaire was created using Google Forms and distributed on social media platforms (Twitter, Telegram, and WhatsApp). The study’s inclusion criteria allowed all adults residing in the western region of Saudi Arabia to participate in the study.

Using an online sample calculator (Raosoft, Raosoft, Inc., Seattle, USA) with a 5% margin of error and a 95% confidence level, the estimated number of required participants was 385. Thus, the 1023 participants that took part in the study were considered to have created a highly representative sample, thereby providing adequate power to prevent type II errors and increase the reliability of the study’s findings.

Questionnaire variables

The questionnaire was formulated based on our study objectives after a search of relevant literature with similar objectives [11-13]. The questionnaire was composed of 19 questions divided into three main domains: demographics, intentional outdoor tanning behaviors, and knowledge of the hazards of UV and tanning. The first domain included questions on age, sex, nationality, education level, and skin type, the answers to which were acquired through the participants’ self-assessment after explanation and classified according to Fitzpatrick’s skin types I to VI. The second domain targeted those participants who practice outdoor tanning and asked questions about their behaviors, including their duration of tanning, sunscreen application during tanning, their recent history of sunburn, and reasons for outdoor tanning. It also asked them to answer the following statement: I think I look better with a tan. The third domain assessed their knowledge of the hazards of UV and tanning based on their answers to the following statements: Sunscreen application is necessary to avoid the harmful effects of the sun; Prolonged sun exposure is dangerous even with the application of high sun protection factor (SPF); Sunburn can occur even when sunlight on the skin doesn’t feel warm; Sun exposure causes the aging, wrinkling, and discoloration of the skin; Sun exposure relates to skin cancer; Tanning is evidence of skin damage; and Light-colored clothing is better protection against the sun than dark-colored clothing. A correct answer scored 1 and an incorrect one scored 0. The total score varied from 0 to 7 points, and they were classified into the following three levels: ≤2 points = poor knowledge, 3-5 points = fair knowledge, and ≥6 points = good knowledge. The final question asked about the source of education on the hazards of UV.

Ethical considerations

This study was approved by the Institutional Review Board at the Directorate of Health Affairs of Jeddah, Ministry of Health, Saudi Arabia (A01440). Before participation in the research, online informed consent was obtained from all the participants.

Statistical analysis

Statistical Product and Service Solutions (SPSS) (IBM SPSS Statistics for Windows, Version 20.0, Armonk, NY) was used to enter and analyze the collected data. The continuous data were reported as mean and SD and analyzed using independent t-tests or one-way analysis of variance (ANOVA) with Bonferroni correction for multiple comparisons as appropriate. The categorical data were reported as frequency and percentages and analyzed using Chi-square tests. P-values < 0.05 (two-sided test) were considered statistically significant.

## Results

A total of 1023 adults were included in the study. The demographics and phenotypes of the study population are presented in Table 1, which shows that 58% of the participants were female and 90% were Saudis. Furthermore, 42.9% of the participants had a Fitzpatrick grade III skin type and the majority of the study population (76.3%) had a bachelor’s and above degree.

**Table 1:** Demographics, level of education, and skin type of study population (n=1023)

Variable		N (%)
Age (year)	18-29	375 (36.7%)
	30-44	373 (36.5%)
	45-59	192 (18.8%)
	60-73	83 (8.1%)
Sex	Female	596 (58.3%)
	Male	427 (41.7%)
Nationality	Saudi	925 (90.4%)
	Non-Saudi	98 (9.6%)
Level of education	Bachelor and above	781 (76.3%)
	Less than bachelor	242 (23.7%)
Fitzpatrick score	Fitzpatrick Type I/II	387 (37.8%)
	Fitzpatrick Type III	439 (42.9%)
	Fitzpatrick Type IV	158 (15.4%)
	Fitzpatrick Type V/VI	39 (3.8%)

**Figure 1:**
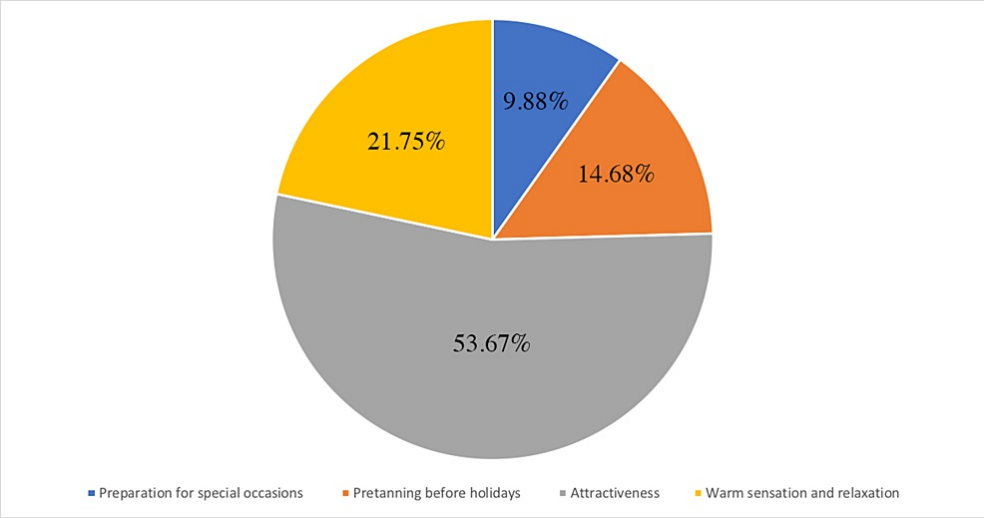
Reasons for outdoor tanning

In addition, outdoor tanning was practiced by 251 of the participants (24.5%), the majority of which (203, 80.9%) exposed themselves to the sun for more than 20 minutes; 54.9% experienced one to four sunburns a year and 76.1% agreed with the statement "I think I look better with a tan" (Table 2). Finally, 53.8% believed that the main reason for outdoor tanning is that it raises the attractiveness of an individual (Figure 1).

**Table 2:** Characteristics of the participants who practice outdoor tanning (n=251)

Variable		N (%)
Duration of tanning	20 minutes or less	48 (19.12%)
	>20 minutes	203 (80.9%)
Sunscreen during tanning	Yes	157 (62.54%)
	No	94 (37.45%)
In the past 12 months how many times did you have a red or painful sunburn that lasted a day or more?	0	93 (37%)
	1-4	138 (54.9%)
	5 or more	20 (7.96%)
I think I look better with a tan	Strongly agree/Somewhat agree	191 (76.1%)
	Neither agree nor disagree	45 (17.2%)
	Strongly disagree/Somewhat disagree	15 (5.97%)

The data in Table 3 show the demographic, skin type, and knowledge of the participants who intentionally tan outdoors and those who do not. The participants who intentionally tan outdoors were significantly younger than those who do not (32 ± 10.7 vs. 38.4 ± 13.9, respectively, P <0.001). Education was associated with practicing outdoor tanning, as it was prevalent among 27.5% of those participants who have a bachelor’s degree or above compared with only 14.9% of those who do not (P <0.001). Neither sex nor nationality was associated with practicing outdoor tanning. Skin type was significantly associated with practicing outdoor tanning and was most prevalent among those participants with skin type IV (30.4%) followed by III (28%), I/II (19.9%), and V/VI (7.7%, P <0.001). Believing that tanning is evidence of skin damage was significantly associated with less practicing of outdoor tanning, as 21.7% of the participants who believe that tanning is evidence of skin damage practiced outdoor tanning compared with 78.3% of those who do not (P = 0.01). The participants who had good knowledge of the disadvantages of sun exposure on the skin practiced outdoor tanning to a lesser extent than those with poor knowledge; however, this association was not statistically significant (P >0.05).

**Table 3:** Demographics, skin type, and knowledge of adults who intentionally tan outdoors and who do not ANOVA: analysis of variance

Variable		Practicing outdoor tanning		P-value
		Yes (n=251)	No (n=772)	
Age (years) (mean ± SD)		32 ± 10.7	38.4 ± 13.9	<0.001
Sex n, (n%)	Female	155 (26%)	441 (74%)	0.196
	Male	96 (22.5%)	331 (77.5%)	
Nationality n, (n%)	Saudi	227 (24.5%)	698 (75.5%)	0.991
	Non-Saudi	24 (24.5%)	74 (75.5%)	
Education n, (n%)	Bachelor and above	215 (27.5%)	566 (75.5%)	<0.001
	Less than Bachelor	36 (14.9%)	206 (85.1%)	
Skin type n, (n%)	I/II	77 (19.9%)	310 (80.1%)	<0.001
	III	123 (28%)	316 (72%)	
	IV	48 (30.4%)	110 (69.6%)	
	V/VI	3 (7.7%)	36 (92.3%)	
Sun exposure causes aging, wrinkling, and discoloration of the skin n, (n%)	TRUE	209 (23.7%)	674 (76.3%)	0.106
	FALSE	42 (30%)	98 (70%)	
Sun exposure relates to skin cancer n, (n%)	TRUE	194 (24.3%)	604 (75.7%)	0.753
	FALSE	57 (25.3%)	168 (74.7%)	
Tan is evidence of skin damage n, (n%)	TRUE	131 (21.7%)	474 (78.3%)	0.01
	FALSE	120 (28.7%)	298 (71.3%)	
Knowledge score n, (n%)	Poor	10 (34.5%)	19 (65.5%)	0.279
	Fair	95 (26%)	271 (74%)	
	Good	145 (23.2%)	480 (76.8%)	
Continuous data is shown as mean and SD. P-value for differences between practicing outdoor tanning groups in continuous data was obtained using one-way ANOVA with Bonferroni correction for multiple comparisons. a denotes significantly different than b. Categorical data is shown as frequency and percentages. P-value for differences between practicing outdoor tanning groups in categorical data was obtained using Chi-square test. Significant differences between knowledge groups are shown in bold font.				

**Figure 2:**
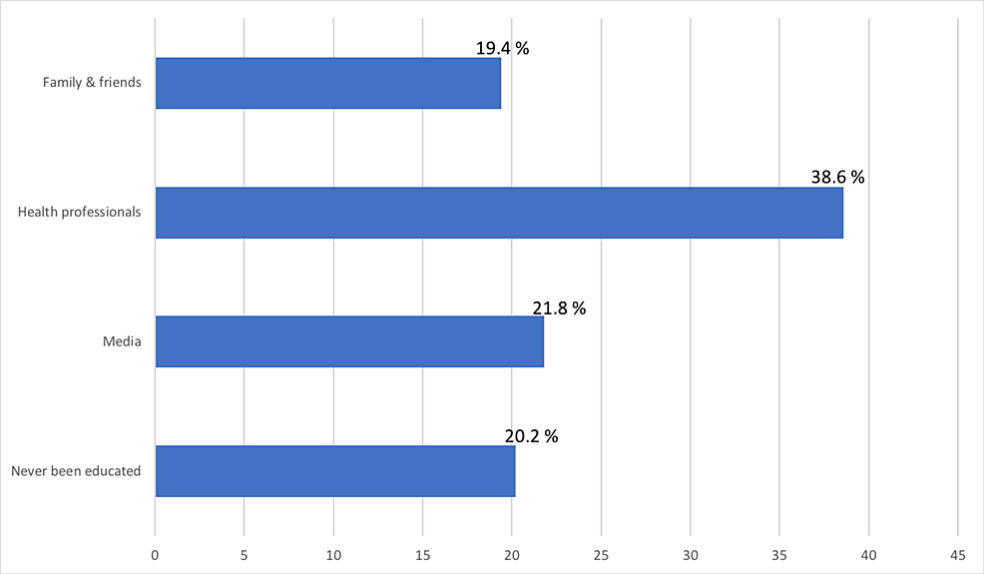
Source of education regarding hazards of UV UV: ultraviolet

Table 4 shows that most of the participants had good knowledge of the disadvantages of sun exposure on the skin (61.3%), followed by fair knowledge (35.9%) and poor knowledge (2.8%). Age was significantly associated with better knowledge. People with good knowledge were significantly younger than those with poor knowledge (35.9 ± 13.2 vs. 42.7 ± 13.1 years, respectively, P = 0.005). In addition, good knowledge was significantly more prevalent in women (70.3%) than men (48.6%) (P <0.001). There was no association between education, nationality, skin type and practicing outdoor tanning, and knowledge of the disadvantages of sun exposure on the skin. Furthermore, the source of education of most of the participants was healthcare professionals, followed by the media and then family and friends (Figure 2).

**Table 4:** Demographics, skin type, and tanning practice in adults with poor, fair, and good knowledge scores ANOVA: analysis of variance

Variables		Knowledge score			P-value
		Poor (n=29)	Fair (n=366)	Good (n=625)	
Age (years) (mean ± SD)		42.7^a^ ± 13.1	37.9 ± 13.8^a,b^	35.9 ± 13.2^b^	0.005
Sex n, (n%)	Female	4 (0.7%)	173 (29%)	419 (70.3%)	<0.001
	Male	25 (5.9%)	193 (45.5%)	206 (48.6%)	
Education n, (n%)	Bachelor and above	23 (2.9%)	278 (35.6%)	479 (61.4%)	0.908
	Less than Bachelor	6 (2.5%)	88 (36.7%)	146 (60.8%)	
Nationality n, (n%)	Saudi	29 (3.1%)	329 (35.6%)	565 (61.2%)	0.201
	Non-Saudi	0 (0%)	37 (38.1%)	60 (61.9%)	
Skin type n, (n%)	I/II	8 (2.1%)	137 (35.5%)	241 (62.4%)	0.766
	III	15 (3.5%)	152 (34.8%)	270 (61.8%)	
	IV	4 (2.5%)	62 (39.2%)	92 (58.2%)	
	V/VI	2 (5.1%)	15 (38.5%)	22 (56.4%)	
Continuous data is shown as mean and SD. P-value for differences between knowledge groups in continuous data was obtained using one-way ANOVA with Bonferroni correction for multiple comparisons. a denotes significantly different than b. Categorical data is shown as frequency and percentages. P-value for differences between knowledge groups in categorical data was obtained using Chi-square test. Significant differences between knowledge groups are shown in bold font.					

## Discussion

In this study, we aim to explore the knowledge and behaviors of intentional outdoor tanning among adults in the western region of Saudi Arabia. Overall, 24.5% of the participants intentionally tanned outdoors. This study finds that outdoor tanning is more prevalent among younger adults. This is consistent with Shoemaker et al.’s study, which found that intentional outdoor tanning was most frequent among young adults [11]. Although previous studies have found a higher prevalence among women, our study did not find significant differences [14,15]. However, we found a higher frequency of outdoor tanning among adults with a higher grade of skin type. This finding is in accordance with the study by Haluza et al. (2016) which revealed a higher prevalence rate of outdoor tanning by those people with darker skin types [16]. By contrast, a study conducted in the United States showed no difference in practicing outdoor tanning by skin type [11].

In regard to taking protective measures such as using sunscreen while tanning, 62.5% of the participants reported using sunscreen compared with 37.4% who denied using it. This could be explained by the common misconception that applying sunscreen reduces the likelihood of achieving a tan. This is a lower percentage than in a study conducted in Germany, which found that 98% of its participants use sunscreen as a protective precaution while on vacation and slightly fewer while tanning 93% [17].

As sunburn could be a sign of DNA damage that, in turn, could increase the risk of skin cancer, it could also indicate not taking protective measures such as the application of sunscreen or even its improper use. We asked the participants about the occurrence of sunburn in the past 12 months and one to four times was the most frequent answer (54.9%). This is similar to a study of adults aged 18-24 years, which observed that 50% had experienced sunburn in the past 12 months [18,19].

Adults who intentionally tanned outdoors believed they looked more attractive. This is consistent with a study in Austria showing that respondents stated attractiveness as the main motivation for outdoor tanning [16]. This is also in accordance with the belief that tanned skin is more attractive than pale skin, perpetrated in society by the media and celebrities.

Unsurprisingly, we observed that women were more aware of the hazards of sun exposure than men (P ≤0.001). This is commonly found given that women are more concerned about their skin health and the photoaging effect caused by sun exposure. A good number of studies in this field have provided evidence in support of this conclusion [20,21].

The source of information was mainly healthcare professionals, followed by the media and then family and friends. This is in contrast to previous studies in which the media was the most influential source of information [22,23]. This demonstrates the huge impact of the media on individuals, which can be used not only to improve knowledge but also to encourage tanning-related healthy attitudes and preventive behaviors rather than glamorizing tanned skin.

The limitations of this study include the cross-sectional nature of the survey and reliance on self-reported data that might not represent tanning trends accurately.

## Conclusions

Individuals who intentionally tan outdoors engage in other behaviors or beliefs that increase their exposure to UV rays. This points to the need for comprehensive interventions such as community-counseling campaigns to address these new trends and their relationship with photoaging and skin cancer.
